# Effect of Violet Light-Transmitting Eyeglasses on Axial Elongation in Myopic Children: A Randomized Controlled Trial

**DOI:** 10.3390/jcm10225462

**Published:** 2021-11-22

**Authors:** Kiwako Mori, Hidemasa Torii, Yutaka Hara, Michiko Hara, Erisa Yotsukura, Akiko Hanyuda, Kazuno Negishi, Toshihide Kurihara, Kazuo Tsubota

**Affiliations:** 1 Department of Ophthalmology, Keio University School of Medicine, 35 Shinanomachi, Shinjuku-ku, Tokyo 160-8582, Japan; morikiwako@gmail.com (K.M.); htorii@keio.jp (H.T.); erisa.yotsuku@icloud.com (E.Y.); akiko-hanyuda@hotmail.co.jp (A.H.); kazunonegishi@keio.jp (K.N.); 2 Laboratory of Photobiology, Keio University School of Medicine, 35 Shinanomachi, Shinjuku-ku, Tokyo 160-8582, Japan; 3 Hara Eye Clinic, 1-5-27 Suehiro, Ohtawara City 324-1142, Tochigi, Japan; yharaohtawara@gmail.com (Y.H.); michiko.h.mama55@icloud.com (M.H.); 4 Tsubota Laboratory, Inc., 304 Toshin Shinanomachi-ekimae Bldg., 34 Shinanomachi, Shinjuku-ku, Tokyo 160-0016, Japan

**Keywords:** violet light, eyeglasses, myopia, axial length, refraction, myopia control, double blinded randomized controlled trial

## Abstract

The fact that outdoor light environment is an important suppressive factor against myopia led us to invent violet light-transmitting eyeglasses (VL glasses) which can transmit violet light (VL), 360–400 nm in wavelength, for the suppression of myopia, and can meanwhile block harmful ultraviolet waves from sunlight. The current study is a double-blinded randomized clinical trial to investigate the myopia-suppressive effect of VL glasses compared to conventional eyeglasses (placebo glasses) that do not transmit VL. The subjects were children aged from 6 to 12 years old, the population in which myopia progression is generally accelerated, and the myopia suppressive effect was followed up for two years in a city in Japan. Periodical ophthalmic examinations, interviews, and measurements of reflection and axial length under mydriasis were performed at the initial visit (the baseline) and at 1, 6, 12, 18, and 24 months. The mean change in axial length in the VL glasses group was significantly smaller than in the placebo glasses group when time for near-work was less than 180 min and when the subjects were limited to those who had never used eyeglasses before this trial (*p* < 0.01); however, this change was not significant without subgrouping. The suppressive rate for axial elongation in the VL glasses group was 21.4% for two years.

## 1. Introduction

Myopia is reported to progress due to both genetic and environmental factors [1], but its precise mechanism remains unclear. Only a few safe and secure preventive measures against myopia progression have been established; in addition, the population suffering from myopia has expanded, exceeding one billion people [2].

When myopia progresses and turns into high myopia, the axial length grows and the shape of the eye changes, which may lead to blindness because of sequelae such as myopic maculopathy, glaucoma, and retinal detachment [3,4]. In a domestic epidemiological study, the Tajimi study, it was shown that high myopia accounted for 20% of all myopia cases and ranked first as the cause of WHO-defined blindness [5]. Additionally, it is reported that one diopter suppression of myopia reduces 20% of the possibility of blindness caused by high myopia [6,7]. In order to avoid blindness, prevention of axial elongation and eye deformation is critically important [8]. Therefore, early intervention to prevent myopia progression is highly significant, as it can considerably reduce the risk of sequelae of high myopia, which may lead to blindness. 

It is crucial to control environmental factors to suppress the progression of myopia. There are some studies regarding environmental factors in relation to myopia progression, such as the Orinda Study [9], the Singapore Cohort Study of the Risk Factors for Myopia [10], and the Sydney Myopia Study [11,12]. These studies revealed that myopia could be accelerated by urban habitation, long-term near work, higher education, and high intelligence quotient (IQ), while outdoor activities suppressed its development. Two hours or more of daily outdoor activity can reduce the onset rate of myopia, irrespective of whether parents are myopic or not, which is one of its genetic factors [13,14]. There were a couple of major RCTs regarding the correlation of outdoor time with myopia. Cao et al. reported the significance of outdoor time for myopia prevention in their systematic review and meta-analysis, based on randomized controlled trials [15]. According to their report, an additional 20 min of recess outside the classroom could help to slow down the change speed of the refractive error [16]. RCTs conducted in China revealed that 40 min of school outdoor activity was added to the outdoor group, and the changes in both refractive error and axial length were slower than those of the control group [17]. A similar finding in another RCT conducted in Taiwan showed similar results [14]. Though it has been considered that the light that is critical for myopia prevention in an outdoor environment is very high intensity of illumination, even low illumination intensity could have myopia suppressive effects on myopia [18]. 

Although many researchers have performed investigations to reveal the reason for the effectiveness of outdoor activities on myopia prevention, there are studies focused on a light wavelength that exists in the outdoor environment. The current environment regarding myopia is characterized by ultraviolet-blocking materials such as windows and eyeglasses [19]. Previous studies have revealed that red, green, blue, and violet have the potential to suppress myopia [20,21,22,23,24,25]; among these wavelengths, violet light (VL: 360–400 nm) is the most potent [26]. Conventional eyeglasses do not penetrate the ultraviolet wavelength, but they also cut off VL [19]. VL eyeglasses were invented to solve these issues and this study was performed to verify their effect. 

There have been some previous studies concerning VL. Torii et al. demonstrated that VL suppressed axial elongation and myopic shift of the refractive error in a lens-induced myopia model using chicks [19]. The same results were demonstrated in other reports using mouse models [24,26]. The mechanism of VL in suppressing myopia progression was revealed to maintain choroidal thickness through OPN5 in the retina [26,27]. OPN5 is an opsin, one of the photoreceptors in the retina, which is sensitive to VL [26]. OPN5 is reported to be associated with the circadian rhythm, vasculogenesis, and thermogenesis [26,28,29]. Another study revealed that EGR1, a myopia-suppressive gene, is associated with VL. EGR1 expression was dominant in the myopia-suppressed enucleated eyes illuminated by VL in chicks [19]. Torii et al. also conducted a retrospective study comparing axial elongation for one year between a partially VL-blocking contact lenses (CL) group, comprising 31 eyes of 31 patients (age range, 13–18 years; mean age, 14.7 ± 1.3 years), and a VL-transmitting CL group comprising 116 eyes of 116 patients (age range, 13–18 years; mean age, 15.1 ± 1.4 years). This study revealed 0.19 mm of mean axial elongation in the partially VL-blocking CL group and 0.14 mm of mean axial elongation in the VL-transmitting CL group (*p* < 0.05) [19]. Another retrospective study revealed that axial elongation in 10 subjects with −6 D or less refractive errors implanted with non-VL-transmitting phakic intraocular lenses (pIOL) was 0.38 mm, and axial elongation in 13 subjects with −6 D or less refractive errors implanted with VL-transmitting pIOL was 0.09 mm for 5 years (*p* < 0.05) [30].

VL exist in the outdoor environments; however, they hardly exist in indoor environments because most of them are blocked by windows [19]. Likewise, VL do not reach our eyes since they are blocked by ordinary eyeglasses [19]. 

Thus, we invented eyeglasses that transmit VL and block harmful short ultraviolet (UV) light from sunlight. We designed the study to investigate the myopia-suppressive effect of our eyeglasses, violet light-transmitting eyeglasses (VL glasses), for two years, comparing with conventional eyeglasses (placebo glasses) that do not transmit VL.

## 2. Materials and Methods

### 2.1. Study Design

This was a prospective randomized double-blind placebo-controlled trial conducted for 2 years. The study was performed in compliance with the Declaration of Helsinki, Ethical Guidelines for Medical and Health Research Involving Human Subjects, and local regulatory requirements, and was also conducted under the approval of all study institutional review boards (IRB) and ethics committees. This trial was approved by the Certified Review Board of Keio (Approval No. N20188004). This trial was also registered by Japan Registry of Clinical Trials with the registration number jRCTs032180418. This randomized control trial followed CONSORT guidelines.

### 2.2. Study Organization

The participants were recruited at Hara Eye Clinic, Tochigi, Japan. The analysis of statistics was outsourced to an independent company (Satista, Inc., Kyoto, Japan), without any relationship with JINS HOLDINGS, Inc., Gunma, Japan, the sponsor of this clinical study, and interpretation after the results of the analysis was performed by the department of ophthalmology and laboratory of photobiology, Keio University School of Medicine, Tokyo, Japan.

### 2.3. Participants and Sample Size

**Figure 1:**
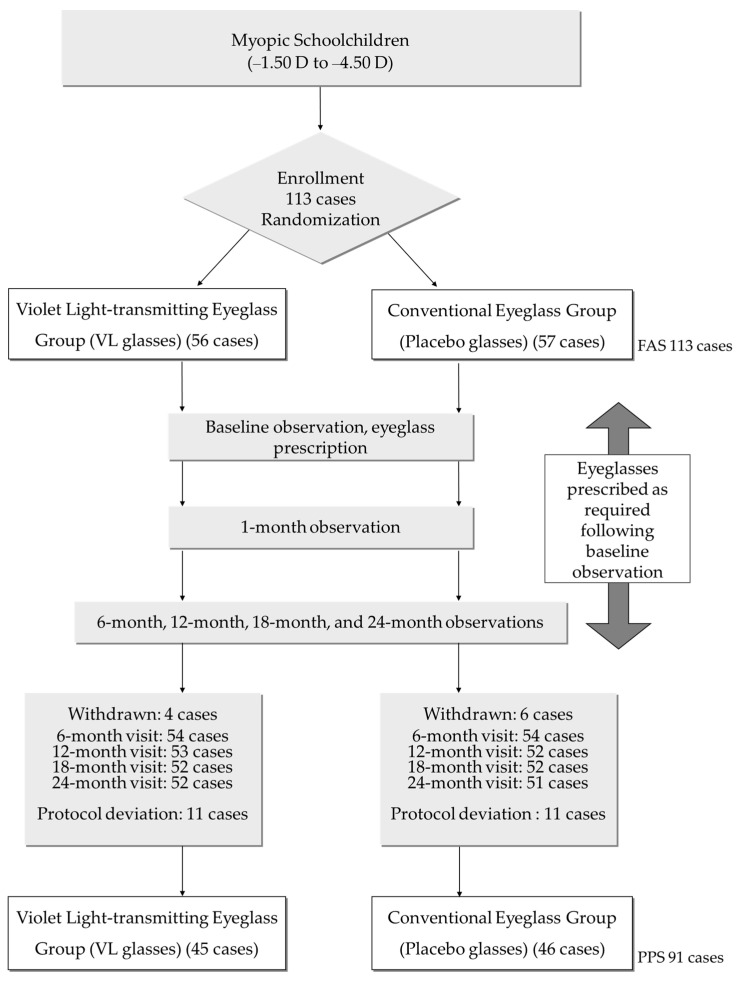
Flowchart of the double-blind randomized clinical trial time points and number of participants. Details of the reasons for withdrawal and protocol deviation are described in Table S1. FAS: full analysis set; PPS: per protocol set.

The participants were enrolled from July 2016 to August 2018 and followed-up for 24 months. As for the sample size, previous research results of MyoVision (Zeiss International) showed values of 0.78 ± 0.29 mm for axial elongation and −1.65 ± 0.80 D for refractive change for two years while wearing conventional eyeglasses [31]. When considering that the suppressive effect of an outdoor environment is 30%, it can be estimated that axial elongation is 0.55 mm and refractive change is −1.16 D for two years. Upon establishing the sample size, along with axial length and refractive change, each group required 34 cases, under the condition that the effect size was 0.23, the standard deviation was 0.29, α = 0.05 (both sides), and 1-β = 0.90 when axial length was the primary outcome. When it was assumed that the dropout rate was 15%, each group required 40 participants, meaning the total sample size would be 80 participants. When refractive change was the secondary outcome, each group required 57 participants, under the condition that the effect size was 0.49, the standard deviation was 0.80, α = 0.05 (both sides), and 1-β = 0.90. Since the refractive change was the secondary outcome in this study, the total sample size was 140 participants when the drop rate was assumed to be 15%. The first participant was enrolled on 17 August 2016. Though the pace of the enrollment was initially steady, it gradually dropped and could not reach the target number by the end of the scheduled recruitment period. Therefore, the recruitment period was extended twice, and the total number of participants was finally 113 (Figure 1). 

Children who met all the criteria were included in the study; (1) those who were aged 6–12 of both gender at the moment of consent; (2) those who spent at least 1 h per a day outdoors; (3) those whose cycloplegic refraction in each eye was between −1.50 D and −4.50 D, (4) those who had one or two parent/s with myopia; (5) those who were able to wear eyeglasses habitually and who could fulfill clinical visits in accordance with the study protocol; (6) those who had no ocular diseases besides ametropia; and (7) those who could provide written informed assent from the study subjects (hereinafter referred to as “subject(s)”) themselves and informed consent from their legal guardian(s). 

Children who met at least one of the following criteria were excluded from the study; (1) those who had worn bifocals or progressive power lenses; (2) those who had worn orthokeratology lenses; (3) those with anisometropia exceeding 1.50 D; (4) those with astigmatism exceeding 1.50 D, (5) those with manifest strabismus; (6) those with a history of refractive surgery; (7) those with a history of keratoconus, herpetic keratitis, or papillary hyperplasia, etc.; (8) those participating in an ongoing similar study; or (9) those who had been judged to be ineligible to participate in the study by the investigators.

### 2.4. Randomization and Masking

Randomization followed the EDC system. Static allocation of stratification by (1) age and (2) gender, i.e., random substitution block method, was performed, and schoolchildren were assigned to either a VL glasses group or a placebo glasses group. The principal investigator and the co-investigator/s were not informed about the details of the allocation steps.

### 2.5. Intervention

**Figure 2:**
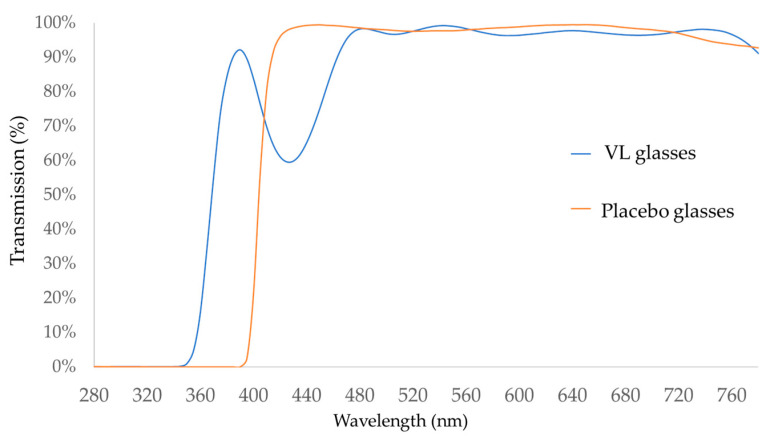
Characteristics of the eyeglasses used in this study: the transmission rate (%) at each wavelength of the light with VL glasses (blue) and with placebo glasses (orange) is shown. VL glasses transmit light 360–400 nm in wavelength, whereas placebo glasses block the light of the wavelengths less than 400 nm.

The intervention group was obliged to wear VL glasses for 24 months, whereas the control group was instructed to wear conventional eyeglasses (placebo glasses) that did not transmit VL (Figure 2). 

### 2.6. Procedure for Follow-Up Examinations

The primary investigator or co-investigator prescribed refraction correcting eyeglasses based on the result of a visual acuity examination under cycloplegia which was conducted at the baseline. Whether the glasses were VL glasses or placebo glasses was not disclosed to the primary or co-investigator at prescription. At the point of regular eye examination, 1, 6, 12, 18, and 24 months after the baseline, over the 24-month research period, new correcting glasses of each type were prescribed to gain 20/20 or more of visual acuity. Barring these points, the same correcting glasses of each type were prescribed only in case of accidental damage or loss of eyeglasses, and the prescriptions were not allowed to be changed. 

At the first encounter, details of the study design and the rights of the participants were explained. An eye examination was performed to measure subjective/objective cycloplegic refraction and axial length following written informed consent. The best corrected visual acuity of subjective refraction was measured under cycloplegia to prescribe the eyeglasses. Objective refraction was measured with a closed-field type auto ref/kerato/tono/pachymeter (TONOREF^®^III, NIDEK, Tokyo, Japan) with 0.01 D increments. The measurement of objective refraction under cycloplegia was performed one hour after the application of 1% cyclopentolate hydrochloride eyedrops (Cyplegin^®^ 1% ophthalmic solution, Santen, Osaka, Japan). Axial length was measured with an IOLMaster 500 (Carl Zeiss Meditec, Jena, Germany). Interviews of the participants were performed at every visit. Participants’ age, gender, number of parents with myopia, living environment, and lifestyles such as time for sunlight exposure, near work, sleep, and physical activities were asked. The time for sunlight exposure and near work was calculated with weighted means of 5 weekdays and 2 weekends. Regular eye examination was performed at 1, 6, 12, 18, and 24 months from the baseline to measure the visual acuity of the prescribed eyeglasses, best corrected visual acuity, subjective refraction, objective refraction under cycloplegia, and axial length. Regarding adverse events, surveillance of each participant during the whole period of this study was performed to report in the form of case reports.

### 2.7. Outcomes

The primary and the secondary outcomes were the change in axial length and objective refraction, i.e., spherical equivalent refraction (SER), under cycloplegia for 24 months, respectively.

### 2.8. Statistical Analysis

All data were analyzed based on the intention-to-treatment principle. The primary analysis was performed in the per-protocol set (PPS), and robustness of the results was explored through sensitivity analysis in the full analysis set (FAS). 

The repeated-measure outcomes were analyzed with a linear mixed-effects model for repeated measures (MMRM) that included intervention, dummy variables for time, intervention-by-time interactions as covariates, and the subjects as a random effect. Furthermore, in this model, all measurements obtained from both eyes were used and entered as repeated effects. The covariance structure was a completely general (i.e., unstructured) covariance matrix. The results were reported as the least squares means with 95% confidence interval (CI) at each time-point. 

The odds ratios (OR) and 95% confidence intervals (CI) were calculated by univariate logistic regression analysis for independent risk factors associated with rapidly progressed myopia, i.e., increase in axial length by 1.2 mm or more, and deterioration of SER by −2.5 D or less at 24 months. Multivariate analysis was performed with adjustment by gender, “already wearing glasses at first visit”, and parental myopia. There are some papers regarding the definition of rapid progression of myopia. Rapid progression of myopia is mostly defined as −1 D or less of decrease in refractive errors per year [32,33]. In the meantime, it is reported that 1.25 D of deterioration of myopia categorized in fast progression of myopia tends to progress more in the following year [34]. In this study, because of the preceding reasons, the degree of −2.5 D or less of progression of myopia for two years was defined as fast/rapid progression of myopia and its exacerbating factor was investigated.

Subgroup analysis according to factors considered to be related to the outcome (i.e., baseline age, already wearing glasses at first visit and baseline time of near-work) was conducted with the MMRM. The time of near-work was calculated by weighted means of 5 weekdays and 2 weekend days.

A *p*-value of <0.05 was considered statistically significant, and all *p*-values were two-sided without multiplicity adjustment. All statistical analyses were performed using SAS 9.4 Foundation for Microsoft Windows for x64 (SAS Institute Inc., Cary, NC, USA).

## 3. Results

### 3.1. Flow of Participants

A total of 113 children were enrolled in this trial. Of these, 57 participants were assigned to the placebo glasses group (placebo group) and 56 participants to the VL glasses group (VL group) (Figure 1). During the follow-up period, 32 participants dropped out; 22 participants deviated from the protocol and 10 withdrew their consent to participate. As a result, a total of 91 participants—46 in the placebo group and 45 in the VL group —completed this trial. The investigators, including orthoptists and ophthalmologists, were masked with regard to the allocation of the groups.

### 3.2. Participant Profiles

The profiles of the participants are shown in Table 1. No significant differences were found between the two groups with respect to age or gender. In addition, SER and axial length at the first visit showed no significant differences. The mean ages of the participants in the placebo and the VL groups were 9.5 ± 1.5 years (mean ± SD) and 9.3 ± 1.5 years; the mean SERs of right eyes were −2.66 ± 0.85 and −2.82 ± 0.87 D; the mean SERs of left eyes were −2.66 ± 0.87 and −2.90 ± 0.92 D; the mean axial lengths of right eyes were 24.53 ± 0.67 and 24.45 ± 0.93 mm, and the mean axial lengths of left eyes were 24.54 ± 0.67 and 24.45 ± 0.97 mm, respectively. No significant differences were found between the two groups except time for near work.

### 3.3. Adverse Events

No adverse effects associated with violet light exposure were reported during the 2-year-clinical study. All adverse events reported during the study were not associated with violet light exposure (Table S2).

### 3.4. Comparison of Myopia Progression after 24 Months 

A total of 113 participants were enrolled and randomly dichotomized into two groups, of which 57 (mean age 9.5 ± 1.5 SD year old, 22 males and 35 females) belonged to the placebo group and 56 (mean age 9.3 ± 1.5 year old, 21 males, 35 females) to the VL group. Finally, 91 participants—46 in the placebo group and 45 in the VL group—were selected after application of exclusion criteria such as familial issues and protocol deviation from the research protocol. PPS is defined as cases excluding subjects who fell into the exclusion criteria. FAS is defined as all the cases included in this study. For example, PPS does not include those who did not spend more than 1 h outdoors (Table S1). In total, 113 subjects were analyzed as FAS and 91 were analyzed as PPS. It was confirmed that randomization was appropriate by analyzing statistics of the background in each group, and the balance of the background was judged to be appropriate. In PPS, the variation in axial length after 24 months was 0.758 mm (95% CI: 0.711–0.810) in the placebo group and 0.728 mm (95% CI: 0.682–0.775) in the VL group, while SER was −1.531 D (95% CI: −1.729–−1.330) in the placebo group and −1.421 D (95% CI: −1.617–−1.225) in the VL group. In the VL group, the average variation in axial length was as small as −0.030 (95% CI: −0.096, 0.037, *p* = 0.381), and that of the spherical equivalent was similarly small at 0.110 (95% CI: −0.168, 0.389, *p* = 0.431), without significant statistical difference by the mixed effect model with individual variation factors and repetition effects of the bilateral eyes (Table 2). 

Factors that contributed to deterioration of myopia were investigated by logistic analysis in this study. This approach suggested that risk factors of 1.2 mm or more of axial elongation were young age, having already worn eyeglasses at the baseline, small change in BMI, paternal myopia, and short sleeping duration (Table 3). Risk factors of −2.5 D or less for SER deterioration were young age, having already worn eyeglasses at the baseline, small change in BMI, and paternal myopia (Table 4). Besides, multivariate analysis was performed with adjustment by gender, “already wearing glasses at first visit”, and parental myopia (Tables S3 and S4). The result of the analysis showed that the odds ratio of “already wearing glasses at first visit” for the deterioration of the axial length and the SER was kept at 4.0 even in the multivariate model. This result suggested that “already wearing glasses at first visit” can be considered to be a deteriorating factor.

When analyzing children limited to those who first started using eyeglasses, 11 children in each group, the change in axial length was 0.856 mm (95% CI: 0.856–1.057) in the placebo group and 0.751 mm (95% CI: 0.646–0.855) in the VL group, respectively, when near-work time was less than 180 min. The change in SER was −1.841D (95% CI: −2.056–−1.626) in the placebo group and −1.538D (95% CI: −1.860–−1.316) in the VL group, respectively. The mean change in axial length in the VL group was significantly small (difference: −0.206 mm; 95% CI: −0.351, 0.060; *p* = 0.006), whereas the mean change in SER in the VL group was small but not significant (difference 0.303, 95% CI: −0.006, 0.612, *p* = 0.055) using a mixed effect model (Table 5, Figure 2).

The results were obtained by linear mixed-effects model analysis. (A) The adjusted means of change in AL in the VL group were significantly (*p* = 0.006) smaller than those in the placebo group at 24 months. (B) The adjusted means of SER changes in the VL group were smaller than those in the placebo group at 24 months, which was not significant (*p* = 0.055). Orange lines show the VL group and blue lines show the placebo group. Error bars show 95% confidence intervals. ** *p* < 0.01. AL: axial length; SER: spherical equivalent refraction; VL group: violet light-transmitting eyeglasses group.

## 4. Discussion

According to previous reports, VL has an effect on suppressing myopia progression [19,26,30]. Based on this research, the application of instruments that could distinguish the effective light to prevent myopia progression from harmful lights to protect the eyes was attempted. The VL glasses, which actually transmit VL and block detrimental constituent such as UV, were invented in our laboratory and were expected to exert potency in clinical situations. This 2-year randomized controlled study was designed to investigate the effectiveness of VL glasses in suppressing the progression of myopia, and it revealed that the mean change in axial length in the VL glasses group was significantly smaller than that in the placebo glasses group when time for near-work was less than 180 min and when the subjects were limited to those who had never used eyeglasses before this trial (*p* < 0.01). This is the first randomized controlled study of VL glasses that reflects their potency. However, this study could not attain statistical significance when no limitation regarding near-work time and eyeglasses histories of the subjects was applied. Because VL transmitting eyeglasses do not exert their effect until they transmit VL in an outdoor environment, it was inappropriate to perform analysis while including the cases who did not have enough time for outdoor activity; therefore, PPS was performed. Nevertheless, since there were unexpectedly many unregistered cases, and those of protocol deviation such as shortage of outdoor activity time, VL glasses were merely found to have a tendency to be effective, but they did not reach statistical significance, even by PPS. The subgroup analysis limited to the group with no history of eyeglasses before this study, and with less than 180 min of near-work time, eventually revealed that VL glasses significantly suppressed axial elongation. The suppressive rate of axial elongation in the VL glasses group for two years was 21.4%, which could be considered meaningful to some extent.

The reason why limiting the subjects with no history of wearing eyeglasses led to the result being significant regarding axial elongation was sought. This study also revealed that the speed of myopia progression in the subgroup that had already worn conventional eyeglasses was actually fast; this result is possibly due to genetic background and the development of myopia at the early stage of life (Table 3 and Table 4). The excessive burden of near-work accelerates myopia progression and may cause attenuation of the effect of VL glasses. In addition, during a period of blocking VL transmission by wearing conventional eyeglasses, myopia progression could be facilitated.

As a prerequisite for a human study, there have been some reports concerning animal experimental models. Exposure to long-wavelength red light developed hyperopic responses in Rhesus monkeys and tree shrews [21,35], whereas red light was, in contrast, demonstrated to induce myopia response in chicks [22]. Meanwhile, short-wavelength light exposure led to hyperopia in chickens, fish and guinea pigs [22,23,25,36]. Furthermore, lens-induced myopia (LIM) models in chicks, mice and guinea pigs showed suppression of axial elongation and myopic shift of refractive error when exposed to VL [19,24,26,37]. Among visible lights, VL was the most effective wavelength for suppressing myopia progression in LIM [26].

VL is characteristic of the shortest wavelength and adjacent to ultraviolet waves. Because of this fact, it has often been considered whether VL is detrimental to the eyes. In this study, we did not find any adverse events during the two-years period through this study by regular examinations, including ocular surface, cataracts, allergy, and the fundus (Table S2). When VL glasses are worn, the amount of VL reaching the eye is more than that when conventional eyeglasses are worn. Furthermore, the amount of VL transmitted when VL glasses are worn is less than that when no glasses are worn. This fact may have contributed to no adverse effects being observed.

**Figure 3:**
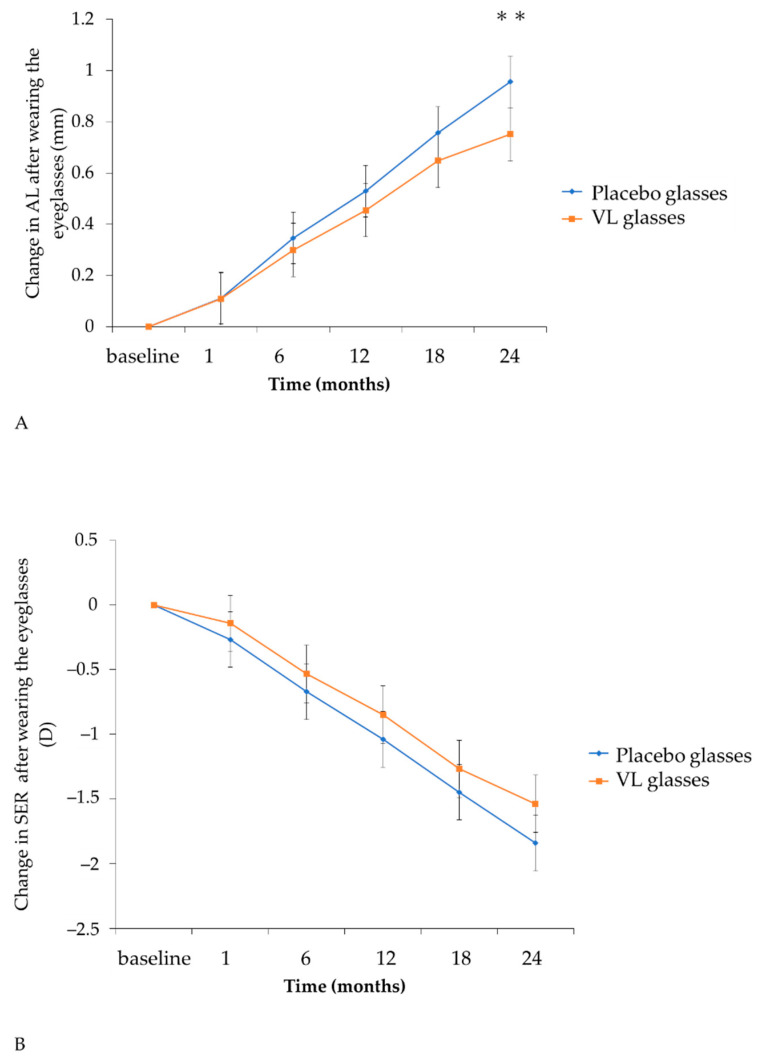
Time course of the adjusted mean axial elongation and SER change under the limited condition in which near-work time was less than 180 min and there was no previous history of eyeglasses use. (**A**) change in AL after wearing the eyeglasses, (**B**) change in SER after wearing the eyeglasses. AL: axial length, SER: spherical equivalent refraction ** *p* < 0.01.

To date, there have been many types of eyeglasses sold to the public. In order to study the pure effectiveness of VL glasses, the subjects were limited to children who had never worn eyeglasses. Moreover, at the baseline, near-work time in the VL group was less than that in the placebo group, as shown in Table 1; it is well known that near-work time is an important factor for the progression of myopia. Therefore, subgroup analysis was performed and was limited to a group in which near-work time was less than 180 min. As a result, axial elongation was suppressed in the VL glasses group unless the time for near-work exceeded 180 min. The suppressive rate of the axial elongation with VL glasses for 2 years was 21.4% (Figure 3A). While this value does not surpass the suppressive rate of axial elongation with orthokeratology, multifocal contact lenses, or the defocus incorporated multiple segments (DIMS) eyeglasses [38,39], it is competitive with other methodologies such as progressive addition lenses (PAL), radial refractive gradient lenses, and positively aspherized PAL. The suppressive rate of axial elongation in PAL was 0–16%, that in radial refractive gradient lenses was not statistically significant, and that in positively aspherized PAL was 12% [31,40,41]. Additionally, the suppressive rate of axial elongation with 0.01% atropine drops, one of the current major standard remedies for myopia suppression, is reported to be 12% in Low-Concentration Atropine for Myopia Progression (LAMP) and 18% in Atropine for the Treatment of Myopia in Japan (ATOM-J) studies [42,43]. VL glasses, the suppressive rate of which is 21.4% under the limited condition regarding near-work time and the history of eyeglasses use, are demonstrated to be barely superior to atropine eye drops as a preventive measure against myopia progression.

This study includes some limitations. First, it was performed in a rural area in Japan where children spend much time outdoors; sufficient outdoor activities were an essential condition to demonstrate the myopia-suppressive effect of the VL glasses, as VL exists in the outdoor environment but not in the indoor environment. However, the result did not follow our expectations. The mean time for outdoor activities in a day did not reach 1 h; therefore, we could not analyze all the participants to judge the effectiveness of the VL glasses. Second, we established the necessary number of subjects by calculating sample sizes referring to previous research regarding MyoVision eyeglasses [31]. Despite our endeavors in recruiting the participants twice and extending the recruitment time, the number of subjects did not reach 140. This is probably because the subjects themselves judged they would waste 2 years when they were assigned to the placebo group, in spite of the existing methods of myopia prevention such as orthokeratology and atropine eyedrops. Moreover, there were many dropout cases during the research period, resulting in analyses of 91 participants as the final number. To make matters worse, the prevalence of COVID-19 disabled the participants in keeping their time for outdoor activities, which influenced the proper analysis regarding the effectiveness of VL glasses in all subjects. These obstacles may have affected the result of this study, not showing statistical significance upon comparing the two groups. Meanwhile, it was considered to be of much importance that subgroup analysis for the participants who had never used any types of eyeglasses before this research revealed the effectiveness of VL glasses on myopia suppression, especially on the suppression of axial elongation. The result of the analysis was not biased by the past usage of any type of eyeglasses, and it truly reflected the potential of the VL glasses. Furthermore, the suppressive effect on myopia of the VL glasses works especially when they are used in the outdoor environments, and therefore, whether the suppressive effect was owing to the VL glasses or the outdoor environments is difficult to discern. However, the outdoor time in the placebo group and that in the VL group were not significantly different. The fact that the suppressive effect of the VL glasses exceeds that of the placebo glasses in the same outdoor time may suggest that myopia progression is due to the difference in whether the glasses transmit VL or not.

## 5. Conclusions

Violet light-transmitting eyeglasses suppressed axial elongation without any adverse events and their suppressive rate was 21.4%.

## 6. Patent

A patent has been applied for the optical components internationally (Patent No. WO2017/090128) and registered in Japan (JP.6629343), US (US.10866433) and China (CN.108474888) by Tsubota Laboratory, Inc. and JINS HOLDINGS, Inc.

## Figures and Tables

**Table 1:** Characteristics of the 113 participants.

Characteristic	Category	All	Placebo	VL	*p* Value	
Number of cases		113	57	56		
Age (years)		9.4 ± 1.5	9.5 ± 1.5	9.3 ± 1.5	0.478	†
Sex	boys	43 (38.1%)	22 (38.6%)	21 (37.5%)	1.000	††
	girls	70 (61.9%)	35 (61.4%)	35 (62.5%)		
Parental myopia	both parents	56 (51.9%)	24 (45.3%)	32 (58.2%)		
	only father	22 (20.4%)	10 (18.9%)	12 (21.8%)	0.196	††
	only mother	30 (17.4%)	19 (35.8%)	11 (20.0%)		
Height (cm)		135.3 ± 10.9	135.8 ± 9.9	134.7 ± 11.9	0.989	†
Weight (kg)		31.16 ± 7.35	31.15 ± 6.86	31.17 ± 7.88	0.629	†
Best corrected visual acuity (log MAR)	right eyes	−0.09 ± 0.03	−0.09 ± 0.03	−0.09 ± 0.03	0.985	
	left eyes	−0.08 ± 0.03	−0.08 ± 0.03	−0.09 ± 0.03	0.718	
Axial length (mm)	right eyes	24.49 ± 0.81	24.53 ± 0.67	24.45 ± 0.93	0.724	
	left eyes	24.50 ± 0.83	24.54 ± 0.67	24.45 ± 0.97	0.658	
SER (D)	right eyes	−2.74 ± 0.86	−2.66 ± 0.85	−2.82 ± 0.87	0.328	
	left eyes	−2.78 ± 0.90	−2.66 ± 0.87	−2.90 ± 0.92	0.156	
Number of participants with glasses at the first visit		59 (52.2%)	28 (49.1%)	31 (55.4%)	0.574	††
**Environmental factors**						
Near-work time (min/day)		193.45 ± 93.13	214.50 ± 104.11	172.02 ± 75.48	0.015	†
Sunlight exposure time (min/day)		58.75 ± 52.18	54.52 ± 47.34	63.03 ± 56.80	0.388	†
Sleeping hours (hours/day)		8.56 ± 0.67	8.57 ± 0.63	8.54 ± 0.72	0.841	†

Data represent means ± SDs; min: minutes; log MAR: logarithm of the minimum angle of resolution; SER: spherical equivalent refraction; D: diopter; VL group: violet light-transmitting eyeglasses group; †: *t*-test; ††: Fisher test; others: Mann–Whitney U test.

**Table 2:** Results of the mixed-effects model fitted to 24-month change for both eyes.

	Placebo			VL			Difference in Amount of Change from the Baseline			
	PPS n = 46			PPS n = 45						
	LS Mean	95% CI		LS Mean	95% CI		Difference	95% CI		*p*-Value
Axial length										
PPS										
first visit (baseline)	24.54	24.31	24.77	24.63	24.4	24.87				
24 months	25.30	25.07	25.53	25.36	25.13	25.59				
change from baseline	0.76	0.71	0.81	0.73	0.68	0.78	−0.03	−0.10	0.04	0.381
SER										
PPS										
first visit (baseline)	−2.73	−2.97	−2.49	−2.96	−3.2	−2.71				
24 months	−4.26	−4.56	−3.96	−4.38	−4.68	−4.08				
change from baseline	−1.53	−1.73	−1.33	−1.42	−1.62	−1.23	0.11	−0.17	0.39	0.434

VL group: violet light-transmitting eyeglasses group; LS mean: least squares mean; 95% CI: 95% confidence interval.; SER: spherical equivalent refraction under accommodative paralysis; PPS: per protocol set. Linear mixed model: variable factors are “individuals,” repeated effects are left and right sides of the participants’ eyes, intervention contents are groups wearing normal glasses and violet light-transmitting glasses, fixed effects are interactions of intervention contents and time.

**Table 3:** Factors for increase in axial length of 1.2 mm or more.

		Univariate Logistic Regression			
		OR	95% CI		*p*-Value
Age (y)		0.28	0.16	0.51	<0.0001
Female		0.56	0.19	1.60	0.279
Change in BMI		0.54	0.30	0.98	0.044
Continuous near-work time (min)		1.00	0.99	1.02	0.643
Continuous near-work time (digital devices) (min)		1.00	0.99	1.02	0.556
Already wearing glasses at first visit		4.67	1.28	17.06	0.020
Near-work time (min)		1.00	0.99	1.00	0.617
Near-work time (digital devices) (min)		1.00	0.99	1.01	0.907
Near-work time (books) (min)		0.99	0.97	1.00	0.171
Outdoor activity time (min)		1.00	0.98	1.01	0.410
Birth weight (kg)		1.00	1.00	1.00	0.403
Birth height (cm)		1.05	0.84	1.32	0.648
Parental myopia					
	Only father	1.00		ref	
	Only mother	0.00	0.00		0.997
	Both parents	0.17	0.05	0.55	0.003
Distance from the television (cm)		0.87	0.48	1.57	0.652
Near-working distance (cm)		1.00	0.93	1.09	0.930
Brightness of the bedroom while sleeping					
	Bright	0.00	0.00		0.999
	Dim	0.73	0.25	2.15	0.570
	Dark	1.00		ref	
Bedtime (hr)		0.38	0.15	0.96	0.041
Sleeping hours (hr)		1.86	0.84	4.09	0.124
Extracurricular activities (outside) (min)		0.60	0.13	2.75	0.507

OR: odds ratio; 95% CI: 95% confidence interval; ref: reference standard; BMI: body mass index.

**Table 4:** Factors for decrease in spherical equivalent power of −2.5 D or less.

		Univariate Logistic Regression			
		OR	95% CI		*p*-Value
Age (y)		0.47	0.31	0.72	0.0004
Female		0.85	0.30	2.37	0.750
Change in BMI		0.35	0.18	0.70	0.003
Continuous near-work time (min)		1.00	0.99	1.02	0.718
Continuous near-work time (digital devices) (min)		1.01	0.99	1.02	0.496
Already wearing glasses at first visit		3.47	1.08	11.13	0.037
Near-work time (min)		1.00	1.00	1.01	0.539
Near-work time (digital devices) (min)		1.00	1.00	1.01	0.189
Near-work time (books) (min)		0.99	0.97	1.00	0.145
Outdoor activity time (min)		0.99	0.98	1.01	0.257
Birth weight (kg)		1.00	1.00	1.00	0.783
Birth height (cm)		1.07	0.85	1.33	0.573
Parental myopia					
	Only father	1.00		ref	
	Only mother	0.28	0.07	1.15	0.077
	Both parents	0.28	0.09	0.90	0.033
Distance from the television (cm)		0.80	0.43	1.49	0.481
Near-working distance (cm)		0.98	0.90	1.06	0.583
Brightness of the bedroom while sleeping					
	Bright	0.00	0.00		0.999
	Dim	1.11	0.37	3.34	0.854
	Dark	1.00		ref	
Bedtime (hr)		0.72	0.32	1.64	0.438
Sleeping hours (hr)		1.13	0.53	2.41	0.761
Extracurricular activities (outside) (min)		0.55	0.12	2.52	0.441

OR: odds ratio; 95% CI: 95% confidence interval; ref: reference standard; BMI: body mass index.

**Table 5:** Results of the mixed-effects model fitted to 24 months of change for both eyes under the limited condition in which near-work time is less than 180 min and there was no previous history of eyeglasses use.

Placebo				VL				Difference in Amount of Change from the Baseline			
n	LS Mean	95% CI		n	LS Mean	95% CI		Difference	95% CI		*p*-Value
Axial length											
Change after wearing the eyeglasses for 24 months											
11	0.96	0.86	1.06	11	0.75	0.65	0.86	−0.21	−0.35	−0.06	0.006
SER											
Change after wearing the eyeglasses for 24 months											
11	−1.84	−2.06	−1.63	11	−1.54	−1.76	−1.32	0.30	−0.01	0.61	0.055

LS mean: least squares mean; 95% CI: 95% confidence interval; SER: spherical equivalent refraction under accommodative paralysis; VL group: violet light-transmitting eyeglasses group. Adjusted by group, time, interaction of group and time, both/left/right eyes.

## Data Availability

All data underlying this article are available in the article and in its online supplementary material. We will willingly share our knowledge, protocol, and expertise when asked.

## Supplementary Materials

The following are available online at https://www.mdpi.com/article/10.3390/jcm10225462/s1, Table S1: Details of the reasons for withdrawn and protocol deviation; Table S2: Safety; Table S3: Factors for increase in axial length of 1.2 mm or more; Table S4: Factors for decrease in spherical equivalent power of −2.5 D or less.
