# Supplementary material for: Effect of Violet Light-Transmitting Eyeglasses on Axial Elongation in Myopic Children: A Randomized Controlled Trial
Source: J Clin Med. 2021 Nov 22;10(22):5462. doi: 10.3390/jcm10225462 (PMC8624215; doi:10.3390/jcm10225462)
Supplement: Supplementary file 1 [file jcm-10-05462-s001.zip › jcm-1423870-supplementary.pdf]

Journal of
Clinical Medicine

Table S1. Details of the reasons for withdrawn and protocol deviation.

Subject	113 cases（male：43 cases, female：70 cases）	
**Withdrawn**	**10 cases**	
	subjects' issue	9
	family issue	1
Protocol deviation	22 cases	including duplication
	not satisfying inclusion criteria 3) those whose cycloplegic refraction in each eye is between -1.50 D and -4.50 D	11
	not satisfying inclusion criteria 4) those who have one or two parent/s with myopia	5
	not satisfying inclusion criteria 2) those who spend outdoor at least 1 hour a day	5
	subjects who simultaneously used other myopia correction	4

Table S2. Safety.

Subject	113 cases（male：43 cases, female：70 cases）	
**Adverse reactions**	**0 case**	
Adverse events	1 case (Not Related)	
	Influenza infection	1

Table S3. Factors for increase in axial length of 1.2 mm or more.

	Univariate logistic regression					Multivariable logistic regression				
	**OR1**	**95% CI**			***P*-value**	**OR2**	**95% CI**			***P*-value**
Age (y)	0.28	0.16	,	0.51	<0.0001					
Female	0.56	0.19	,	1.60	0.279	0.73	0.24	,	2.21	0.573
Already wearing glasses at first visit	4.67	1.28	,	17.06	0.020	4.23	1.13	,	15.78	0.032
Parental myopia										
only father	1.00		ref			1.00		ref		
only mother	0.00	0.00	,		0.997					
both parents	0.17	0.05	,	0.55	0.003	0.47	0.15	,	1.48	0.198

OR: odds ratio; 95% CI: 95% confidence interval; ref: reference standard; BMI: body mass index; OR1: non-adjusted, OR2: adjusted by gender, already wearing glasses at first visit and parental myopia.

Table S4. Factors for decrease in spherical equivalent power of −2.5 D or less.

	Univariate logistic regression					Multivariable logistic regression				
	**OR1**	**95% CI**			***P*-value**	**OR2**	**95% CI**			***P*-value**
Age (y)	0.47	0.31	,	0.72	0.0004					
Female	0.85	0.30	,	2.37	0.750	0.95	0.33	,	2.72	0.921
Already wearing glasses at first visit	3.47	1.08	,	11.13	0.037	3.55	1.09	,	11.49	0.035
Parental myopia										
only father	1.00		ref			1.00		ref		
only mother	0.28	0.07	,	1.15	0.077					
both parents	0.28	0.09	,	0.90	0.033	0.51	0.17	,	1.47	0.210

OR: odds ratio; 95% CI: 95% confidence interval; ref: reference standard; BMI: body mass index; OR1: non-adjusted, OR2: adjusted by gender, already wearing glasses at first visit and parental myopia.
